# Inclusion Compound of Efavirenz and γ-Cyclodextrin: Solid State Studies and Effect on Solubility

**DOI:** 10.3390/molecules26030519

**Published:** 2021-01-20

**Authors:** Susana Santos Braga, Firas El-Saleh, Karyna Lysenko, Filipe A. Almeida Paz

**Affiliations:** 1 LAQV-REQUIMTE, Department of Chemistry, University of Aveiro, 3810-193 Aveiro, Portugal; karynalysenko@ua.pt; 2 Ashland Specialty Ingredients, Paul-Thomas Strasse, 56, D-40599 Düsseldorf, Germany; FElSaleh@ashland.com; 3 Department of Chemistry, CICECO—Aveiro Institute of Materials, University of Aveiro, 3810-193 Aveiro, Portugal; filipe.paz@ua.pt

**Keywords:** cyclodextrin inclusion, solution-phase, solid state, antiretroviral

## Abstract

Efavirenz is an antiretroviral drug of widespread use in the management of infections with human immunodeficiency virus type 1 (HIV-1). Efavirenz is also used in paediatrics, but due to its very poor aqueous solubility the liquid formulations available resort to oil-based excipients. In this report we describe the interaction of γ-cyclodextrin with efavirenz in solution and in the solid state. In aqueous solution, the preferential host–guest stoichiometry was determined by the continuous variation method using ^1^H NMR, which indicated a 3:2 host-to-guest proportion. Following, the solid inclusion compound was prepared at different stoichiometries by co-dissolution and freeze-drying. Solid-state characterisation of the products using FT-IR, ^13^C{^1^H} CP-MAS NMR, thermogravimetry, and X-ray powder diffraction has confirmed that the 3:2 stoichiometry is the adequate starting condition to isolate a solid inclusion compound in the pure form. The effect of γ-cyclodextrin on the solubility of efavirenz is studied by the isotherm method.

## 1. Introduction

Efavirenz (EFV) is a potent antiretroviral of widespread use as first-line therapy for patients with HIV infection. Administered in the form of a tablet and requiring only one dose per day [1], efavirenz is a practical therapeutic option. It is classified by the Biopharmaceutical Classification System (BCS) as a class II drug, that is, it is poorly water soluble and highly permeable [2]. Several approaches to improve the solubility of efavirenz are reported, from the manipulation of polymorphs [2] and blending with superdisintegrants [3] or into solid dispersions [4,5], to a broad variety of encapsulation strategies, which include liposomes [6], micelles [7,8], lipidic nanoparticles [9,10], and polymeric nanoparticles [11].

**Figure 1:**
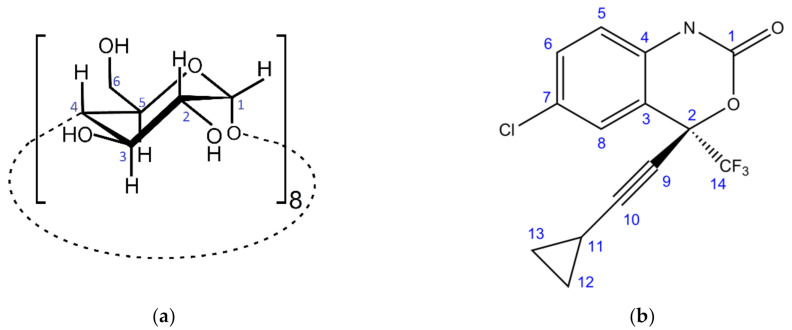
Chemical structure and atom labelling of (**a**) γ-cyclodextrin; (**b**) efavirenz.

Molecular encapsulation of efavirenz with cyclodextrins is a less explored but quite promising solution for the amelioration of the physicochemical and organoleptic properties of this drug. Besides having low aqueous solubility, efavirenz presents a very bitter taste, and for this reason it may benefit from the taste-masking effect resulting from inclusion of each efavirenz molecule into the cavity of the cyclodextrin host. Cyclodextrins are cyclic oligosaccharides formed by natural or biotechnological enzymatic action on starch. The most abundant native cyclodextrins occur with six (α-CD), seven (β-CD), or eight (γ-CD, Figure 1a) D-glucose units, linked together by α-1,4 glycosidic bonds. The unique molecular geometry of cyclodextrins, with the shape of a truncated cone having the secondary hydroxyl groups facing the wider rim and the primary hydroxyls at the narrower rim, while the cavity is lined with protons, gives them hydrophobicity at the cavity in tandem with good aqueous solubility [12,13]. Cyclodextrins are thus used as biocompatible, solubilising, stabilising, and taste-masking agents for a variety of drugs [14], with highlight to the antiviral remdesivir [15] and to ozalin, a recently approved paediatric sedative and the only known commercial drug to contain γ-CD in its composition, acting as a solubiliser and taste-masking agent [16]. Cyclodextrins are gaining increased interest in medicinal applications, being employed as co-adjuvants in vaccines [17] or even as experimental drugs in disorders associated with lipid buildup, as Niemann–Pick disease or focal segmental glomerulosclerosis [18].

Efavirenz is, thus far, only known to form inclusion complexes with two chemical derivatives of beta-cyclodextrin: HPβCD (randomly (2-hydroxy)propylated β-CD) and RAMEB (randomly methylated β-CD); inclusion into the native β-CD was also investigated but the formation of an authentic inclusion complex in the solid state was not demonstrated [19]. The study, conducted by Sathigari et al., further showed that the freeze-dried adducts with RAMEB and HPβCD increased efavirenz solubility in water (at 180 min) from 5.64 ± 1.149% (pure drug) to 54.25 ± 1.031% and 43.13 ± 0.331%, respectively. In a preliminary approach to the inclusion of efavirenz into native cyclodextrins, we investigated the possibility of using β-CD and γ-CD as hosts for efavirenz [20]. Efavirenz and each cyclodextrin were co-dissolved in a water/alcohol solution and the mixture was co-precipitated by cooling; in the batch of efavirenz with β-CD, the precipitate consisted of crystals of the two separate components [20], which confirmed that β-CD does not have adequate cavity size for efavirenz. Our results were, therefore, in good agreement with the findings of Sathigari et al. [19]. In the batch of efavirenz and γ-CD, a new, low-crystalline phase was formed, indicating the formation of an inclusion complex [20]. The preliminary findings on the formation of γ-CD·EFC prompted further investigation on this host-guest system, which is herein presented. Studies were conducted both in the aqueous solution phase and in the solid state. Results indicate the presence of a complex with 3:2 host–guest stoichiometry, in both the solution and solid phase.

## 2. Results and Discussion

### 2.1. Stoichiometry in a Water–Methanol Solution

Inclusion stoichiometry in solution was first assessed as a means to understand the preferences of this host–guest system. For this, ^1^H spectra were collected for series of solutions of EFV and γ-CD, prepared by the continuous variation method (see Section 3.3 for details).

**Figure 2:**
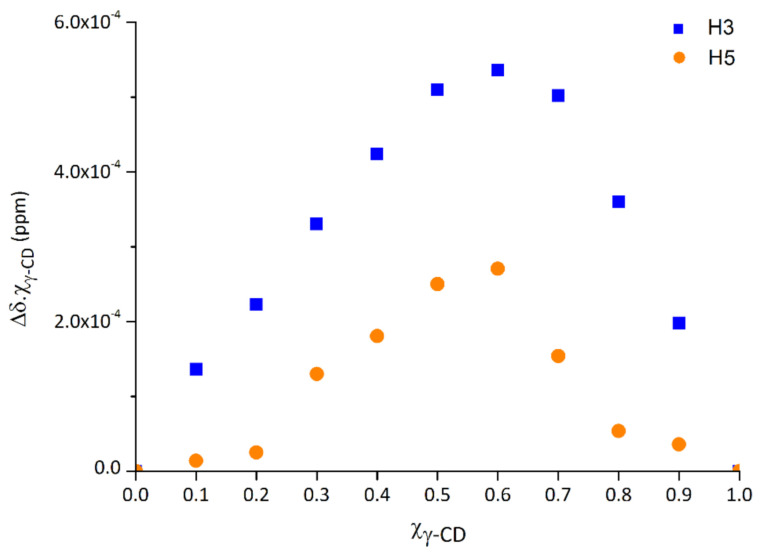
Job plot of the host protons H3 and H5 for the inclusion of efavirenz into γ-CD, measured in a mixed solution of D_2_O and CD_3_OD (1:1).

The plot of χ(γ-CD) × Δδ against the χ(γ-CD), for the two protons of γ-CD located at the inner cavity (H3 and H5), is represented in the Figure 2. The maximum of the experimental points, indicative of the host-to-guest proportion in the inclusion complex, is found at the χ(γ-CD) value of 0.6, thus indicating that the preferred stoichiometry is 3:2, that is, the most abundant species in this liquid medium is (γ-CD)_3_·(EFV)_2_. Note that the presence of other species, namely the 1:1 complex, i.e., (γ-CD)·(EFV), albeit in lower abundance, cannot be excluded [21,22,23].

### 2.2. Solid-State Studies

Procedures for the preparation of inclusion complexes of efavirenz and γ-CD as solid materials comprised combining two separate solutions of each component. Efavirenz, dissolved in ethanol, was mixed with γ-CD, dissolved in ultrapure water, to obtain a clear mixed solution that was subsequently subjected to snap-freezing and freeze-drying. In agreement with the information obtained from the studies in solution, two different host-to-guest stoichiometries were tested, 3:2 and 1:1.

#### 2.2.1. FT-IR Spectroscopy

**Figure 3:**
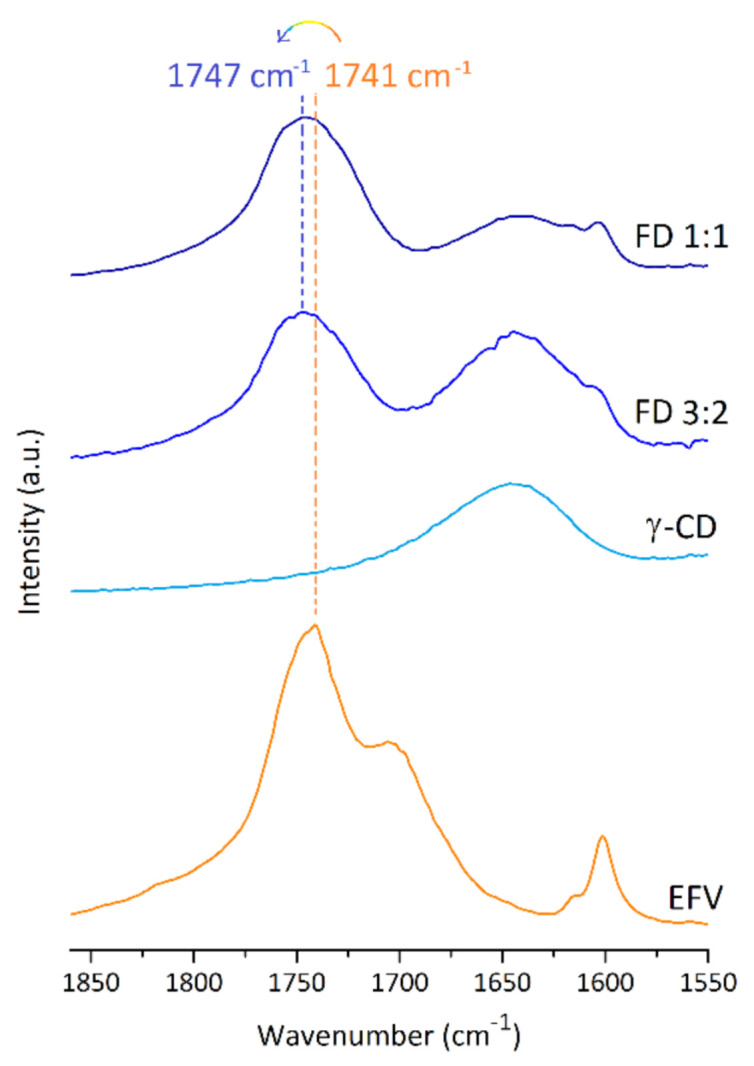
Fourier-transform infrared spectra of efavirenz (EFV), γ-CD and the two freeze-dried solid products starting from mixed solutions with γ-CD:EFV proportions of 3:2 and 1:1 (FD).

Infrared spectroscopy can provide a quick insight into the formation of inclusion complexes, in particular for guests that contain, like efavirenz, oscillators sensitive to the hydrophobic environment of the host cavity and located in a spectral area free from host bands (which could eventually superimpose with it). The carbonyl (C=O) group of efavirenz is thus an excellent probe for inclusion. The carbonyl stretching frequency occurs at 1741 cm^−1^ in the spectrum of pure efavirenz, appearing blueshifted and with maxima centred at 1751 and 1746 cm^−1^ in the spectra of the 3:2 and the 1:1 products, respectively (Figure 3). A blueshift, i.e., an increase in the stretching energy of an oscillator, can be the result of the lower polarisation of the C=O group, with increased electron density in the double bond. It is frequently the result of an apolar environment around the affected oscillator, such as that caused by inclusion into the cavity of γ-CD. For the 1:1 product, the smaller blueshift can be interpreted as the combined result of two contributions, one from the inclusion complex and a second one originating from a small contamination with non-included efavirenz.

#### 2.2.2. Powder X-ray Diffraction

**Figure 4:**
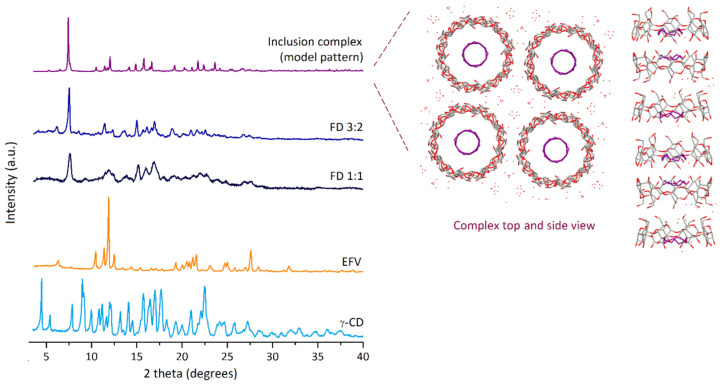
Experimental powder X-ray diffractograms of γ-CD, efavirenz (EFV), and the freeze-dried samples (FD) with γ-CD:EFV stoichiometries of 1:1 and 3:2 (rehydrated prior to data collection to increase their crystallinity). For comparison it also shows the trace of the inclusion complex γ-CD·12-crown-4-ether [24], calculated from its atomic coordinates using Mercury 3.5.1 (Copyright CCDC 2001–2014). The inset depicts the structure of γ-CD·12-crown-4-ether, as viewed from the top (crystallographic *c* axis) and from the side (*a* axis); the molecules of the crown ether guest are represented in purple for differentiation from those of the γ-CD macrocycle.

Powder X-ray diffraction (PXRD) is a very useful tool in identifying the formation of inclusion complexes. It also contributes to investigate the presence of any eventual impurities that may appear, such as crystallites of the host or the guest. PXRD data is presented in Figure 4. Efavirenz presents a diffractogram with several well-resolved peaks that are indicative of its high crystallinity. The most intense reflections occur at 6.3, 10.5, 11.4, 11.9, 12.6, 19.3, 20.0, 20.5, 20.8, 21.2, 21.6, 23.0, 24.7, 25.0 and 27.6 degrees of 2θ.

A first attempt at collecting diffractograms of the two freeze-dried products revealed them to be mostly amorphous (results not shown), which was expected as a result of the preparation method. Restoration of the hydration waters in these samples was performed as a means to increase their crystallinity. The process consisted in placing the bulk materials at ambient temperature in a water-saturated atmosphere during ca. 16 h. PXRD patterns of the rehydrated freeze-dried compounds present overall quasi-similar diffraction patterns and they comprise essentially a new phase, with no traces of crystallites of γ-CD heptahydrate. The diffractogram of the sample with 3:2 stoichiometry exhibits reflections peaking at 5.3, 6.1, 7.5, 8.6, 10.7, 11.5, 12.3, 13.7, 14.2, 15.0, 15.7, 16.1, 16.6, 16.9, 18.9, 20.1, 20.9, 21.6, 22.2 and 22.6 degrees of 2θ. For the compound prepared with a starting stoichiometry of 1:1 (sample FD 1:1 in the Figure 4), the diffractogram is less well resolved, with the main reflections presenting some broadening and peaking at 7.6, 11.5, 11.9, 12.3, 13.7, 15.0, 16.0 and 16.9 degrees of 2θ. It should be noted that the peak centred around 11.9 degrees in the latter sample, albeit poorly resolved, coincides with the most intense reflection of pure efavirenz, thus indicating some degree of contamination with non-included efavirenz (similarly to the observations made from FT-IR data).

Figure 4 also shows the calculated diffractogram of γ-CD∙12-crown-4-ether [24], which is herein used as a representative model for the only known isostructural series of γ-CD inclusion complexes [25]. The diffractogram of the 3:2 freeze-dried sample shows an overall diffraction envelope that is similar to that of the model complex, which suggests it belongs to this isostructural series [25]. It is, thus, fair to assume the inclusion complex of γ-CD and EFV should present the host molecules stacked in infinite channels, similarly to the host organisation reported for the complexes of this isostructural series and herein exemplified for γ-CD∙12-crown-4-ether (inset in Figure 4).

#### 2.2.3. ^13^C{^1^H} CP-MAS NMR

**Figure 5:**
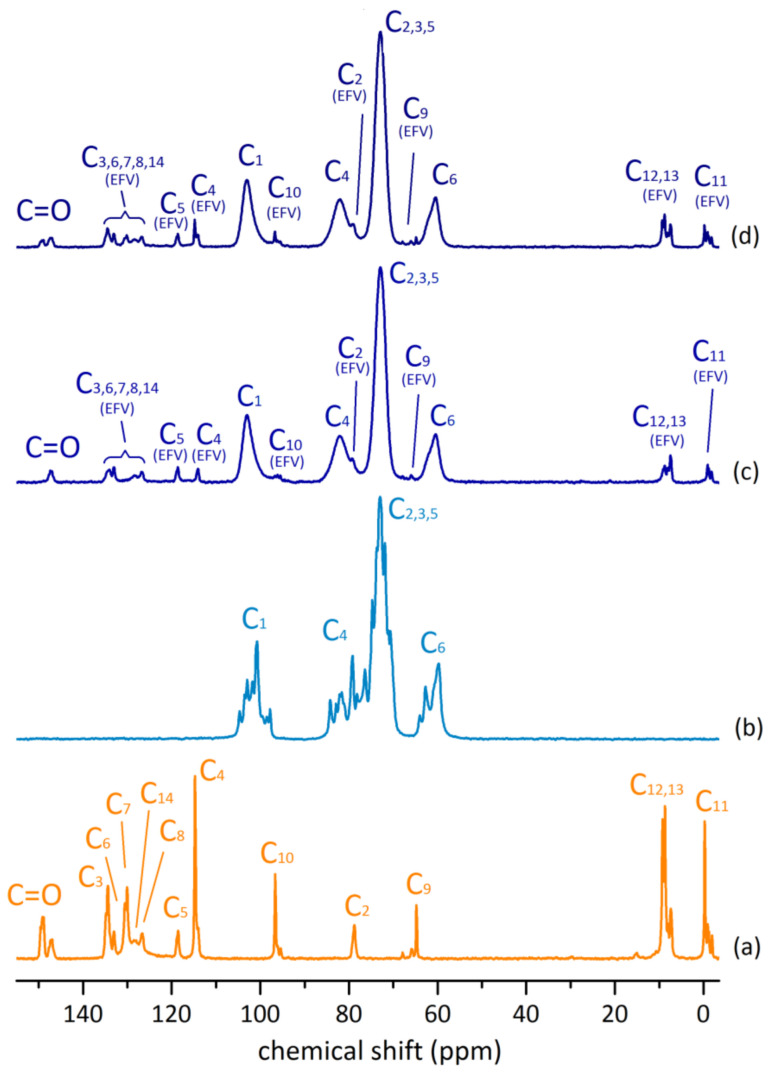
^13^C{^1^H} CP-MAS NMR spectra of (**a**) EFV, (**b**) γ-CD, (**c**) (γ-CD)_3_·(EFV)_2_, and (**d**) γ-CD·EFV (see labelling in Figure 1). Efavirenz carbons were assigned as in Rodrigues de Sousa et al. [26].

The solid-state NMR spectra of efavirenz, γ-CD heptahydrate and the two freeze-dried products of γ-CD with EFV are depicted in Figure 5. 

The spectrum of efavirenz presents a set of well-resolved resonances, with multiple signals observed for its carbons with the exception of C_2_. Signal multiplicity for EFV carbons was previously reported by Rodrigues de Sousa et al., having been attributed to the presence of more than one polymorphs of efavirenz and to different molecular conformations [26]. The host, γ-CD, exhibits multiple sharp resonances for each type of carbon atom, which is ascribed, for C_1_ and C_4_ carbons, to differences in the conformation about the α-1,4 bonds, and, for carbons located closer to the rims, as is the case of C_6_, to ambient changes in the hydrogen-bonding network and the varying number of hydration water molecules [27,28]. In the spectra of (γ-CD)_3_·(EFV)_2_ and γ-CD·EFV, the host carbons appear as single broad resonances, thus indicating symmetrisation of the γ-CD as a result of inclusion of EFV and of the spatial organisation into channels. Regarding the guest signals, and as previously noted for FT-IR results, the 1:1 sample, γ-CD·EFV, shows contamination with pure efavirenz. In ^13^C{^1^H} CP-MAS NMR, this is particularly evident when one observes the carbonyl region. The spectrum of γ-CD·EFV exhibits two resonances for the carbonyl (C_1_, see Figure 1) with chemical shift similar to those of pure efavirenz, thus indicating the presence of non-included guest. In turn, the spectrum of (γ-CD)_3_·(EFV)_2_, bears only one resonance for the carbonyl. This indicates the presence of the pure inclusion complex, in which inclusion into the cavity of γ-CD resulted in a more symmetrical chemical environment for the C=O.

#### 2.2.4. Thermogravimetric Analysis

**Figure 6:**
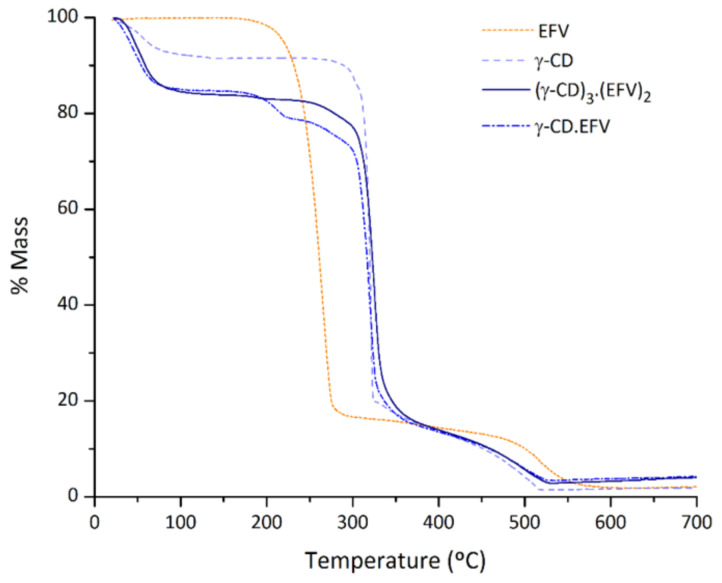
Thermogravimetry traces for efavirenz, γ-CD, and the freeze-dried adducts (γ-CD)_3_·(EFV)_2_ and γ-CD·EFV.

The thermograms of efavirenz, γ-CD heptahydrate and the two freeze-dried products, (γ-CD)_3_·(EFV)_2_ and γ-CD·EFV, are represented in Figure 6. The thermogram of pure efavirenz shows no mass losses from ambient temperature until about 185 °C, temperature that marks the onset of its decomposition. The absence of mass losses at temperatures lower than 100–130 °C indicates the absence of hydration waters, which is expectable due to the apolar nature of this compound. In turn, the thermogram of γ-CD heptahydrate is marked by an initial dehydration step that starts at ambient temperature and proceeds up to 95 °C. This step features a mass loss of 9% that translates into seven hydration waters, being thus coherent with the original specifications. 

The thermogravimetric traces of the two freeze-dried products are marked by an increase in the number of hydration waters. This is evidenced by a more intense dehydration step, rounding 15.5%. For the 3:2 complex, the results allow inferring a general formula of (γ-CD)_3_·(EFV)_2_·(H_2_O)_39_. The increase in the number of hydration waters in comparison to those exhibited by the host is a characteristic of γ-CD inclusion complexes and it results from their distinctive tetragonal symmetry. As described in the section referring to PXRD, γ-CD molecules pack, with the guest molecules lodged inside the channel cavity and also with the formation of wide inter-channel spaces that are able to retain a large number of water molecules (see inset in Figure 4). Literature examples of γ-CD complexes with a large number of hydration waters include γ-CD·quercetin·(H_2_O)_17_ [29], γ-CD·fisetin·(H_2_O)_17_ [30], and (γ-CD)_3_·(resveratrol)_4_·(H_2_O)_62_ [31], to name only a few.

The presence of a strong dehydration step in the trace corresponding to the 1:1 sample is also indicative of the presence of an inclusion complex, however, the mass loss observed between ca. 180 and 220 °C denotes contamination with some amount of pure efavirenz that decomposes at this temperature. It is important to highlight that the step associated with efavirenz thermal decomposition is absent from the thermogram of (γ-CD)_3_·(EFV)_2_, which provides definitive corroboration of the presence of the pure inclusion complex in this sample.

### 2.3. Effect of γ-CD on Efavirenz Solubility

**Figure 7:**
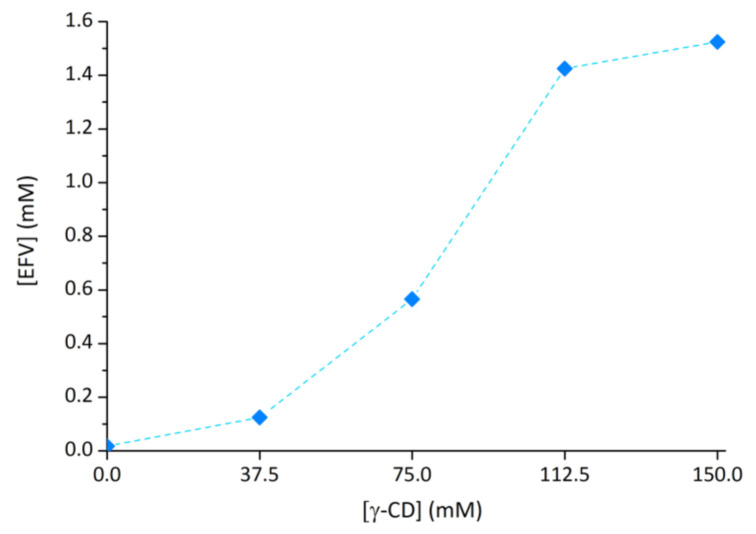
Phase solubility isotherm for efavirenz (EFV) in the presence of γ-CD.

The solubilising effect of medicinal applications of γ-CD on efavirenz was evaluated by collecting the solubility isotherm for this API while in the presence of increasing concentrations of the host. Results are shown in Figure 7. The aqueous solubility reported for efavirenz is very low, with values between 0.0127 mM [32] and 0.0263 mM [33]. The isotherm data herein shown demonstrates that the presence of 37.7 mM γ-CD in solution increases the solubility of efavirenz to 0.124 mM, that is, by at least 5-fold. Efavirenz solubility increases further with the increase in concentration of γ-CD in aqueous solution, with 0.565 mM of EFV dissolved when γ-CD concentration is 75.0 and 1.424 mM of EFV dissolved for the γ-CD concentration of 112.5 mM. After this point, a plateau is reached, indicating the formation of aggregates containing only γ-CD molecules. Self-aggregation of γ-CD molecules is well known, and it can be attributed to their symmetry and extensive intermolecular hydrogen bonding.

For a brief discussion of these results, it is worth comparing them with those of β-CD, HPβCD, RAMEB and HPγCD, previously reported as solubilisers for EFV. The effect of RAMEB on EFV solubility was equivalent to that of γ-CD, with 80 mM of RAMEB solubilising roughly 0.5 mM of this guest [19]. Nevertheless, RAMEB is only approved for topical use (at the nasal and ocular mucosa), which limits its interest as a solubilising agent [14]. Regarding HPβCD, the literature shows contradictory data, one study depicting it as a good solubiliser, with 60 mM increasing EFV solubility to roughly 1 mM [19], whereas our previous report, in which the isotherm data were collected under the same conditions as those of the present study, revealed it to perform worse than γ-CD, since a concentration of 125 mM of HPβCD was required to solubilise ca. 0.5 mM of EFV [20]. Our study also evaluated HPγCD, which had an isotherm similar to that of HPβCD up to 150 mM and performed slightly worse at higher concentrations [20].

## 3. Materials and Methods

### 3.1. Materials

Pharmaceutical-grade γ-CD (Cavamax W8 Pharma) from Wacker-Chemie was kindly donated by Ashland Specialty Ingredients (Düsseldorf, Germany). Efavirenz was obtained from Smillax Pharma (Heiderabad, India).

Ultrapure water was used for the inclusion procedures. All organic solvents were of analytical grade, except otherwise specified.

### 3.2. Equipment

Laboratory powder XRD data were collected at ambient temperature on an Empyrean PANalytical diffractometer (Cu Kα_1,2_ X-radiation, λ_1_ = 1.540598 Å; λ_2_ = 1.544426 Å) equipped with an PIXcel 1D detector and with the sealed tube operating at 45 kV and 40 mA (Bruker AXS, Karlsruhe, Germany). Intensity data were collected by the step-counting method (step 0.01°), in continuous mode, in the ca. 3.5 ≤ 2θ ≤ 50° range.

Solution-phase ^1^H nuclear magnetic resonance (NMR) spectra were recorded on an Avance 300 spectrometer (Bruker Biospin, Rheinstetten, Germany) at 300.13 MHz, at ambient temperature. A 50:50 solution of deuterated water and deuterated methanol was used as solvent, with the residual proton signal of methanol (^1^H 3.31 ppm) and tetramethylsilane (TMS) being used as internal references. The chemical shifts are quoted in parts per million.

^13^C{^1^H}CP/MAS NMR spectra were recorded at 100.62 MHz on a (9.4 T) Avance III 400 spectrometer (Bruker Biospin), with an optimised π/2 pulse for ^1^H of 4.5 μs, 3 ms contact time, a spinning rate of 12 kHz, and 4 s recycle delays. The chemical shifts are quoted in parts per million from TMS.

Infrared spectra were obtained as KBr pellets in a 7000 FTIR spectrometer (Mattson, Oakland, CA, USA) (resolution 2.0 cm^−1^; 128 scans per spectrum).

TGA studies were performed on a Shimadzu TGA-50 thermogravimetric analyser (Kyoto, Japan), using a heating rate of 5 C min^−1^, under air atmosphere, with a flow rate of 20 mL min^−1^. The sample holder was a 5 mm ø platinum plate and the sample mass was about 5 mg.

UV-Vis data for the solubility isotherms were collected on a spectrophotometer Analytik Jena Specord 200 Plus.

### 3.3. Continuous Variation Method

The continuous variation method reported by Job [34] provides an estimate of the preferred stoichiometry in solution based on the measured changes in a physical parameter (in the present case, the chemical shift of sample protons). A series of solutions were prepared using as solvent a 1:1 mixture of deuterated water and deuterated methanol. In each solution, the ratio of host and guest, *r*, varied in steps of 0.1 while their sum (γ-CD]_0_ + [EFV]_0_) was kept constant, at a value of 0.01 M. For the host, γ-CD, *r* is thus defined as:

*r*_γ-CD_ = [γ-CD]_0_/{[γ-CD]_0_ + [EFV]_0_}) (1)

### 3.4. Preparation of the Inclusion Complexes as Solid Materials

#### 3.4.1. γ-CD with EFV in the 3:2 Stoichiometry

A solution of γ-CD (213.1 mg, 0.15 mmol) in ultrapure water (1.5 mL) at 40 °C was treated with another solution of EFV (31.5 mg, 0.10 mmol) in ethanol (0.2 mL). The mixed solution was stirred for 3 min and then subjected to snap-freezing in liquid nitrogen. Solvents were subsequently removed by freeze-drying to obtain a white solid.

FT-IR: ν(tilde) (cm^−1^) = 3405 s, 2934 m, 2894 sh, 2254 w, 1751 m, 1647 m, 1502 m, 1456 sh, 1417 m, 1385 m, 1373 m, 1337 m, 1302 m, 1252 m, 1200 m, 1161 s, 1102 sh, 1080 s, 1054 sh, 1027 vs, 1002 s, 944 m, 933 m, 864 w, 830 vw, 760 m, 743 w, 707 m, 693 w, 656 w, 610 w, 585 m, 531 w, 482 w.

^13^C{^1^H} CP-MAS NMR: δ (ppm) = 147.3 (EFV C_1_), 134.4 (EFV C_3_), 133.1 (EFV C_6_), 130.0 (EFV C_7_), 128.4 (EFV C_14_), 126.8 (EFV C_8_), 118.7 (EFV C_5_), 114.2 (EFV C_4_), 103.1 (γ-CD C_1_), 96.1 (EFV C_10_), 82.2 (γ-CD C_4_), 79.3 (EFV C_2_), 73.0 (γ-CD C_2,3,5_), 66.0 (EFV C_9_), 60.7 (γ-CD C_6_), 8.8, 7.3 (EFV C_12,13_), −0.3, −0.9, −1.7 (EFV C_11_) ppm.

#### 3.4.2. γ-CD with EFV in the 1:1 Stoichiometry

A solution of γ-CD (142.0 mg, 0.10 mmol) in ultrapure water (1.5 mL) at 40 °C was treated with another solution of EFV (31.5 mg, 0.10 mmol) in ethanol (0.2 mL). The mixed solution was stirred for 3 min and then subjected to snap-freezing in liquid nitrogen. Solvents were subsequently removed by freeze-drying to obtain a white solid.

FT-IR: ν(tilde) (cm^−1^) = 3390 s, 2932 m, 2893 sh, 2252 w, 1746 m, 1637 m, 1499 m, 1458 m, 1414 m, 1383 m, 1372 m, 1335 m, 1302 m, 1249 m, 1198 m, 1187 m, 1159 s, 1099 sh, 1079 s, 1052 sh, 1025 vs, 1000 s, 942 m, 931 sh, 861 w, 838 vw, 760 m, 742 w, 706 m, 691 w, 674 vw, 671 vw, 655 w, 608 w, 580 m, 566 sh, 528 w, 480 w

^13^C{^1^H} CP-MAS NMR: δ (ppm) = 149.2, 147.3 (EFV C_1_), 134.5 (EFV C_3_), 133.1 (EFV C_6_), 130.2 (EFV C_7_), 128.4 (EFV C_14_), 126.8 (EFV C_8_), 118.7 (EFV C_5_), 114.9, 114.2 (EFV C_4_), 103.1 (γ-CD C_1_), 96.8, 95.6 (EFV C_10_), 82.2 (γ-CD, C), 79.1 (EFV C_2_), 73.0 (γ-CD C_2,3,5_), 66.1, 64.9 (EFV C_9_), 60.8 (γ-CD C), 8.8, 7.5 (EFV C_12,13_), −0.1, −0.8, −1.7 (EFV C_11_) ppm.

For comparison, the data for efavirenz is as follows:

FT-IR data of efavirenz: ν(tilde) (cm^−1^) = 3255 s, 3183 s, 3094 m, 3020 w, 2951 w, 2876 w, 2253 s, 1894 vw, 1741 vs, 1705 s, 1602 s, 1498 vs, 1457 m, 1428 w, 1403 m, 1134 m, 1060 m, 1333 vs, 1280 s, 1251 vs, 1184 vs, 1168 vs, 1096 s, 1073 s, 1028 s, 976 s, 948 s, 931 s, 884 m, 866 m, 835 m, 824 s, 755 m, 742 m, 711 s, 689 s, 668 w, 656 m, 568 m, 556 m, 541 m, 518 w, 484 m, 460 w, 417 w.

^13^C{^1^H} CP-MAS NMR of efavirenz: δ (ppm) = 149.3, 147.5 (C_1_), 134.5 (C_3_), 133.2 (C_6_), 130.7, 130.2 (C_7_), 128.4 (C_14_), 126.8 (C_8_), 118.8 (C_5_), 114.8, 114.2 (C_4_), 96.8, 95.5 (C_10_), 79.0 (C_2_), 66.0, 64.9 (C_9_), 9.4, 8.9, 8.1, 7.5 (EFV C_12,13_), −0.1, −0.8, −1.0, −1.8 (EFV C_11_).

### 3.5. Solubility Isotherms

Excess amounts of EFV were added to 20 mL of unbuffered aqueous solutions of increasing concentrations of γ-CD. Solutions were stirred for 48 h at room temperature. Aliquots were filtered (0.22 µm), diluted with a 1:1 mixture of water and isopropanol and their absorbance was measured at 293 nm.

## 4. Conclusions

The results described in the present report demonstrated that inclusion of efavirenz into γ-CD occurred both in solution and in the solid state, forming a complex with 3:2 stoichiometry, that is, (γ-CD)_3_·(EFV)_2_. The bulky nature of efavirenz, with a quasi-planar central bicyclic benzoxazin-2-one ring, and two substituents, a cyclopropylethynyl and a trifluoromethyl, protruding laterally from the main plane of the rings, implies that a host with a large cavity is required for molecular encapsulation. It is noteworthy that, even though we employed γ-CD, a host with a wide cavity diameter, inclusion required 1.5 host units per each molecule of efavirenz—the host-to-guest stoichiometry of 3:2 is quite rare for γ-CD inclusion complexes.

Powder X-ray diffraction further evidenced that the complex belongs to the isostructural series of γ-CD inclusion complexes with tetragonal symmetry in which the molecules of γ-CD are stacked in infinite channels with the guest molecules located inside. This contributed to the symmetrisation of the environment around the carbons of efavirenz, particularly C1, which is part of a carbonyl group, and that was observed as a single resonance in solid-state NMR and as a blueshifted vibrational band in FT-IR. The solubilising effect of γ-CD over the efavirenz guest was evaluated by collection of the solubility isotherm for this host–guest system to reveal a B_s_-type diagram [35], that is, the association with γ-CD increases EFV solubility but only to a certain point, which is followed by a plateau. Besides the solubilising action of γ-CD on efavirenz, its ability to mask the bitter taste of this drug is another attractive application for the inclusion complex that warrants demonstration in future studies.

## Data Availability

Solid-state characterisation data is available upon request from the NMR and diffraction services of the University of Aveiro.

## Abbreviations

API	Active pharmaceutical ingredient
CP-MAS	Cross-polarisation with magic angle spinning (a solid-state NMR method)
FT-IR	Fourier-transform infrared spectroscopy
γ-CD	Gamma-cyclodextrin
HPβCD	(2-hydroxy)propyl-beta-cyclodextrin
HPγCD	(2-hydroxy)propyl-gamma-cyclodextrin
EFV	Efavirenz
NMR	Nuclear magnetic resonance
ppm	Parts per million
PXRD	Powder X-ray diffraction
RAMEB	Randomly methylated beta-cyclodextrin
TGA	Thermogravimetric analysis
TMS	Tetramethylsilane
